# High fructose induces dysfunctional vasodilatation via PP2A-mediated eNOS Ser1177 dephosphorylation

**DOI:** 10.1186/s12986-022-00659-3

**Published:** 2022-03-24

**Authors:** Jiaqi Jin, Jingya Liu, Yong Luo, Hong He, Xinyue Zheng, Chaoyang Zheng, Yi Huang, Yang Chen

**Affiliations:** 1 School of Pharmaceutical, Guangzhou University of Chinese Medicine, No. 232 Waihuan Dong Rd., Guangzhou University Town, Panyu District, Guangzhou, 510000 China; 2 Department of Obstetrics and Gynecology, Key Laboratory for Major Obstetric Diseases of Guangdong Province, The Third Affiliated Hospital of Guangzhou Medical University, No. 63 Duobao Road, Liwan District, Guangzhou, 510150 China; 3 Department of Pharmacology, School of Basic Medicine, Tongji Medical College, Huazhong University of Science and Technology, Wuhan, 430030 Hubei China; 4 Department of Cardiology, The Second Clinical Medical College and Guangdong Provincial Hospital of Chinese Medicine, Guangzhou University of Chinese Medicine, Guangzhou, 510006 China; 5 Department of Stomatology, The First Affiliated Hospital, The School of Dental Medicine, Jinan University, No. 613W. Huangpu Avenue, Guangzhou, 510630 China

**Keywords:** Fructose, Dysfunctional vasodilatation, NO, eNOS, PP2A

## Abstract

**Background:**

Processed foods are popular and contain large amounts of industrial fructose, which changes people’s diet and exacerbates the negative health effects of high fructose. Several studies have shown that excessive intake of fructose has a major impact on vascular disease. However, the mechanism of the effect of high fructose on blood vessels is currently unclear.

**Methods:**

The effect of fructose on the vasodilatation of isolated thoracic aortic rings was observed by using wire myography in wild-type (WT) mice. Cell viability and nitric oxide (NO) production were assessed by the corresponding kits in mouse vascular endothelial cells. The effect of fructose on endothelial nitric oxide synthase (eNOS) and protein phosphatase 2A (PP2A) and their changes in phosphorylation were detected by using Western blots. Moreover, a PP2A inhibitor (okadaic acid, OA) was used to evaluate the relationship between fructose and PP2A. Furthermore, PP2ACα endothelial-specific knockout (PP2A cKO) mice were used to detect the vasodilatation of in vitro fructose-incubated thoracic aortic rings by using wire myography.

**Results:**

High fructose induced endothelium-dependent dysfunctional vasodilatation. High fructose reduced acetylcholine (Ach)-induced vasodilation but did not affect sodium nitroprusside (SNP)-induced vasodilation. Accordingly, NO production and the phosphorylation level of eNOS at serine (Ser) 1177 (P-eNOS) in vascular endothelial cells were remarkably reduced without changes in cell viability. The expression of protein phosphatase 2A catalytic subunit (PP2AC) was increased and the expression of phosphorylated PP2AC (P-PP2A, tyrosine [Tyr] 307) was significantly decreased. Nevertheless, these effects were reversed by OA. Moreover, knockout of the PP2A gene could recover the response of vessels to Ach under high fructose stimulation.

**Conclusions:**

Our observations demonstrate an underlying mechanism of fructose-induced dysfunctional vasodilatation. Fructose could activate PP2A, which leads to decrease in the phosphorylation of eNOS at Ser1177 and the reduction of NO release, thus leading to the occurrence of endothelium-dependent dysfunctional vasodilatation.

## Introduction

Industrial fructose has become a sweetener widely used in food and beverage processing and, is different from natural fructose. Fructose in natural foods is related to its vitamins, minerals and fibres, suggesting that these foods contain other healthy nutrients [1]. However, fructose in industrially processed foods often has few nutrients, and this "bad fructose" is the main source of fructose. Fructose intake has increased significantly with the improvement of people's living standards, mostly among teenagers and young adults [2]. However, several studies have noted that high fructose has a greater adverse health effect than high glucose. High fructose is more likely to increase the levels of de novo lipogenesis, saturated fatty acids, total cholesterol, triglycerides, uric acid, and low-density lipoprotein [3–5], which are all predisposing factors of cardiovascular disease. A series of epidemiological studies have shown that people wlho consume excessive fructose have an increased the probability of cardiovascular disease [6, 7], and the prevalence of cardiovascular disease is associated with an exponential increase in industrial fructose intake [8]. Cardiovascular disease is the leading cause of mortality [9]; moreover, patients are gradually showing a "younger" trend [10], which is cause for concern [11].

Vascular injury is an important cause of cardiovascular diseases [12]. It is well known that blood vessels have a certain degree of elasticity, and the function of blood vessels is usually evaluated according to contraction or relaxation [13]. When dysfunctional vasodilatation occurs, it will lead to chronic heart failure [14], atherosclerosis [15], hypertension [16], and other cardiovascular diseases in severe cases. Therefore, preventing dysfunctional vasodilatation is also the key to the prevention of cardiovascular disease. Endothelial cells cover the inner surface of all blood vessels and form a single layer of cells that are most directly in contact with blood [17]. Endothelial dysfunction is a hallmark of many cardiovascular diseases. Previous studies have shown that high glucose [18], angiotensin II [19], and lipopolysaccharide [20] could induce endothelial dysfunction. Vascular endothelial cells play a crucial role in regulating vascular tone. Studies have found that these cells can secrete a variety of endothelium-derived relaxing factors and endothelium-dependent hyperpolarization factors to regulate and control vascular tone [21]. When the production of relaxation factors derived from the endothelium is reduced, it will lead to endothelial dysfunction, which is the first step of cardiovascular diseases [22].

The phosphorylation system plays an essential role in the physiological and pathological process of endothelial dysfunction. The phosphorylation system includes phosphorylation and dephosphorylation, both of which are regulated by a large number of kinases, such as protein kinase C, adenosine monophosphate activated protein kinase, and phosphatases, such as protein phosphatase 2A (PP2A) and protein phosphatase 1. [23]. PP2A is a major serine/threonine phosphatase responsible for dephosphorylation. Its dysregulation is common in many diseases, including cardiovascular diseases, neurodegenerative diseases and cancer [24]. A study reported that high glucose activates PP2A in endothelial cells, leading to apoptosis, dysfunction and inflammation of endothelial cells [25]. The activity of PP2A is affected by its phosphorylation level. When the phosphorylation level at the tyrosine [Tyr] 307 site increased, its activity decreased [26]. Endothelial nitric oxide synthase (eNOS) is a phosphorylated protein that is mainly expressed in endothelial cells and is the only rate-limiting enzyme in the formation of NO from L-arginine [27]. eNOS is phosphorylated at serine, threonine and tyrosine residues, and studies have found that the most important phosphorylation sites occur on serine residues [28]. The activity of eNOS could be enhanced by phosphorylation of the C-terminal serine residue (e.g., Serine [Ser] 1177 of human eNOS), and dephosphorylation could limit the normal production of NO [29]. Although studies have shown that fructose could affect vessel function and eNOS protein expression [30], the mechanism by which fructose triggers eNOS change is not fully understood.

Based on the above, we hypothesized that fructose may affect the endothelial phosphorylation system. This study focuses on how fructose downregulates the phosphorylation level of eNOS to reduce NO release, leading to the occurrence of dysfunctional vasodilation.

## Materials and methods

### Chemicals and instruments

Dulbecco's modified Eagle’s medium (DMEM) (11995056), 0.25% trypsin/ethylenediaminetetraacetic acid (EDTA) (2520056) and foetal bovine serum (16140071) were purchased from Gibco, United States. BCA (P0028), NO (S0021), and loading buffer (P0015L) were purchased from Biyuntian Company, China. CCK-8 assays (KN868) were purchased from Dojindo Company, Japan. Phenylephrine (PE) (61-76-7) and acetylcholine (Ach) (60-31-1) were purchased from Sigma Company, United States. Sodium nitroprusside (SNP) (13755-389) and magnesium sulphate heptahydrate (10034-99-8) were purchased from Macklin Company, China. Sodium chloride (7647-14-5), potassium dihydrogen phosphate (7778-77-0), glucose (14431-43-7), and calcium chloride (10043-52-4) were purchased from Guangzhou Chemical Reagent Factory, China. Fructose (3589), dimethylsulfoxide (DMSO) (3869), potassium chloride (3724), and sodium bicarbonate (3510) were purchased from Tianjing Damao Chemical Reagent Factory, China. EDTA (60-00-4) was purchased from Coolaber, China. Protease inhibitor (04693132001) was purchased from Roche Company, Switzerland. Polyvinylidene fluoride (PVDF) membranes (ISEQ00010) were purchased from Millipore Company, United States. Skim milk powder (232100) and RIPA buffer (G3424) were purchased from GBCBIO, China. Phosphate buffer solution powder (AR0030) and β-actin (1:1000, BM0627) antibodies were purchased from Boster Company, China; eNOS (1:1000, 32027), PP2AC (1:1000, 2038S), anti-mouse (3:5000, 4408S), anti-rabbit (1:1500,7076S) were purchased from CST, United States; P-PP2A (PP2ACα, Tyr 307; 1:1000, AF4453) and P-eNOS (Ser 1177; 1:1000, AF3247) were purchased from Affinity, China. Genotyping primers were purchased from TaKaRa, Japan. PCR mix (P2011) was purchased from Dongsheng Biotech, China. Agarose (TSJ00010) was purchased from Tsingke, China.

### Animals

Male wild type (WT) C57BL/6 J mice (6–9 weeks old) were purchased from Beijing Vital River Laboratory Animal Technology Co., Ltd. (production licence: SCXK 2019–0001). PP2ACα endothelial-specific knockout (PP2A cKO) mice were bred from PP2ACα^flox/flox^ mice, Tie2 mice, and Tie2 mice containing Cre recombinase in vascular endothelial cells. PP2ACα^flox/flox^ mice were provided by Zhejiang University, and Tie2 mice were obtained from the South China Research Center for Acupuncture and Moxibustion (GZUCM, Guangzhou, China).

Mice were housed with water and food ad libitum in a 12 h/12 h reverse light/dark cycle at a constant temperature of 20–22 °C. All protocols were approved by the Institutional Animal Care and Use Committee of Guangzhou University of Chinese Medicine (Guangzhou, China).

### Genotyping

For confirmation of the PP2ACα gene, genotyping was performed by PCR analysis of DNA isolated from mouse tails. The following genotyping primers were used:

Cre: 1084: (5′-GCGGTCTGGCAGTAAAAACTATC-3′);

1085: (5′-GTGAAACAGCATTGCTGTCACTT-3′);

7338: (5′-CTAGGCCACAGAATTGAAAGATCT-3′);

7339: (5′-GTAGGTGGAAATTCTAGCATCATCC-3′).

Loxp: Ca-2-F: (5′-TAGCCCATGCCTTTAATCTCAGAGC-3′).

Ca-2-R: (5′-CACTCGTCGTAGAACCCATAAACC-3′).

PCR amplification procedure setting:

Cre: DNA was denatured at 95 °C for 3 min, and then denatured at 94 °C for 30 s. The annealing step was at 51.7 °C for 1 min and extended at 72 °C for 1 min. After 35 cycles, DNA strands were extended at 72 °C for 5 min and stored at 4 °C.

Loxp: DNA was denatured at 94 °C for 2 min and then denatured at 94 °C for 30 s. The annealing step was at 55 °C for 30 s and extended at 72 °C for 30 s. After 35 cycles, DNA strands were extended at 72 °C for 2 min and stored at 4 °C.

The PCR products were analysed by 1.5% agarose after electrophoresis (110 V, 45 min). A gel image system (Tanon-2500, Tianneng Technology Corporation, China) was used to collect images.

### Measurement of vascular tone

Mice were anaesthetized with isoflurane. The thoracic aorta was isolated through an opening in the abdomen after collecting blood from the posterior orbital venous plexus and placed in physiological salt solution containing 95% O_2_ and 5% CO_2_ (NaCl, 130 mM; KCl, 4.7 mM; KH_2_PO_4_, 1.18 mM; NaHCO_3_, 14.9 mM; MgSO_4_·7H_2_O, 1.17 mM; glucose, 5.5 mM, and CaCl_2_,1.6 mM). The aorta was cleansed of excess connective tissue and fat and cut into rings (2–3 mm in length). Next, the vascular rings were incubated in either 0.25 mM or 2 mM fructose solution for 0.5 h in vitro. Measurement of vascular tone were recorded by wire myograph (Myo Technology A.S, Denmark). Before the experiment, the basal tension of each ring was adjusted to 5 mN until equilibration. KCl (60 mM) was given twice for stimulation to prove the standardization of the vascular rings. After elution to reach equilibrium, 10^–9^ ~ 10^–5^ M Ach was given to observe the vasodilation effect of Ach on 10^–6^ M PE-precontracted without elution. The same method was used to observe the vasodilation effect of SNP on blood vessels. The process was carried out at 37 °C, 95% O_2_ and 5% CO_2_.

### Cell culture and treatment

The microvascular endothelial cell line was originally isolated from mouse hemangioendothelioma and purchased from ATCC (CRL-2586). Microvascular endothelial cells (MVECs) were cultured in DMEM, containing 10% FBS and 1% penicillin–streptomycin. The cells were cultured in a humidified incubator at 37 °C with 5% CO_2_ and 95% air. Cells were passaged by trypsin/EDTA once every two days, and the plate was seeded after cell counting and used for the experiment after adherence. Fructose was dissolved in phosphate buffer solution to prepare a 1 M stock solution, diluted to 0.25, 0.5, 1, 2, 3, and 4 mM and added to the cells when appropriate. A PP2A inhibitor (okadaic acid, OA) was first dissolved in DMSO to prepare a stock solution, diluted to 20 nM when used, and added 1 h before fructose was administered.

### Measurements of cell viability and nitric oxide (NO)

Ishimoto and colleagues have shown that the serum fructose concentration of normal mice could reach approximately 0.25 mM [31]. Therefore, 0.25 mM fructose was used as the control in the experiment. The MVECs were divided into five groups according to the concentration of fructose (0.25, 0.5, 1, 2, 4 mM) and were given the corresponding amount of fructose for 24 h. The experiment was strictly carried out according to the instructions of the CCK-8 kit, and absorbance was measured at 450 nm to detect cell viability. In the NO measurement experiment, the cells were divided into four groups (0.25, 0.5, 1, 2 mM fructose), and were given the corresponding concentration of fructose and/or 20 nM OA for 4 and/or 24 h. Cell culture supernatant was collected, the operation followed the instructions of the NO kit strictly, absorbance was measured at 540 nm, and the standard curve was drawn to calculate NO production. All data were collected by a microplate reader (Thermo Fisher, United States).

### Western blot analysis

The MVECs were divided into four groups (0.25, 0.5, 1, 2 mM fructose), and given the corresponding amount of fructose and/or 20 nM OA to stimulate 8 h. Total protein was extracted using RIPA buffer containing protease inhibitor, and the concentration was measured with the BCA kit. The cell homogenate was adjusted to be consistent with loading buffer and denatured for 5 min at 98 °C. Sodium dodecyl sulphate–polyacrylamide gel (8%) was used to separate the protein at 100 V and transferred onto PVDF membranes at 100 V for 2 h. Five percent milk or BSA was chosen for blocking for 2 h according to the antibody instructions. The corresponding primary antibody was added and incubated for 1 h at room temperature, and then, the membranes were transferred to 4 ℃ and incubated overnight. On the second day, the membranes were incubated in anti-rabbit/mouse for 2 h at room temperature. A chemiluminescence imaging analysis system was used to collect images and analyse the grey value by ImageJ (NIH, United States).

### Statistical analysis

Data were processed by GraphPad Prism 8.0 (GraphPad Software, Inc., USA), and the experimental results are expressed as the mean ± SEM. In the vascular reactivity experiment, the diastolic rate was calculated as the percentage of Ach or SNP diastolic and PE-precontraction values. GraphPad Prism was used to fit the dose–response of the agonist to a sigmoidal curve. Student’s t test was used to evaluate the difference between two groups, and *P* < 0.05 was considered statistically significant.

## Results

### High fructose directly induces endothelium-dependent dysfunctional vasodilation

**Fig. 1:**
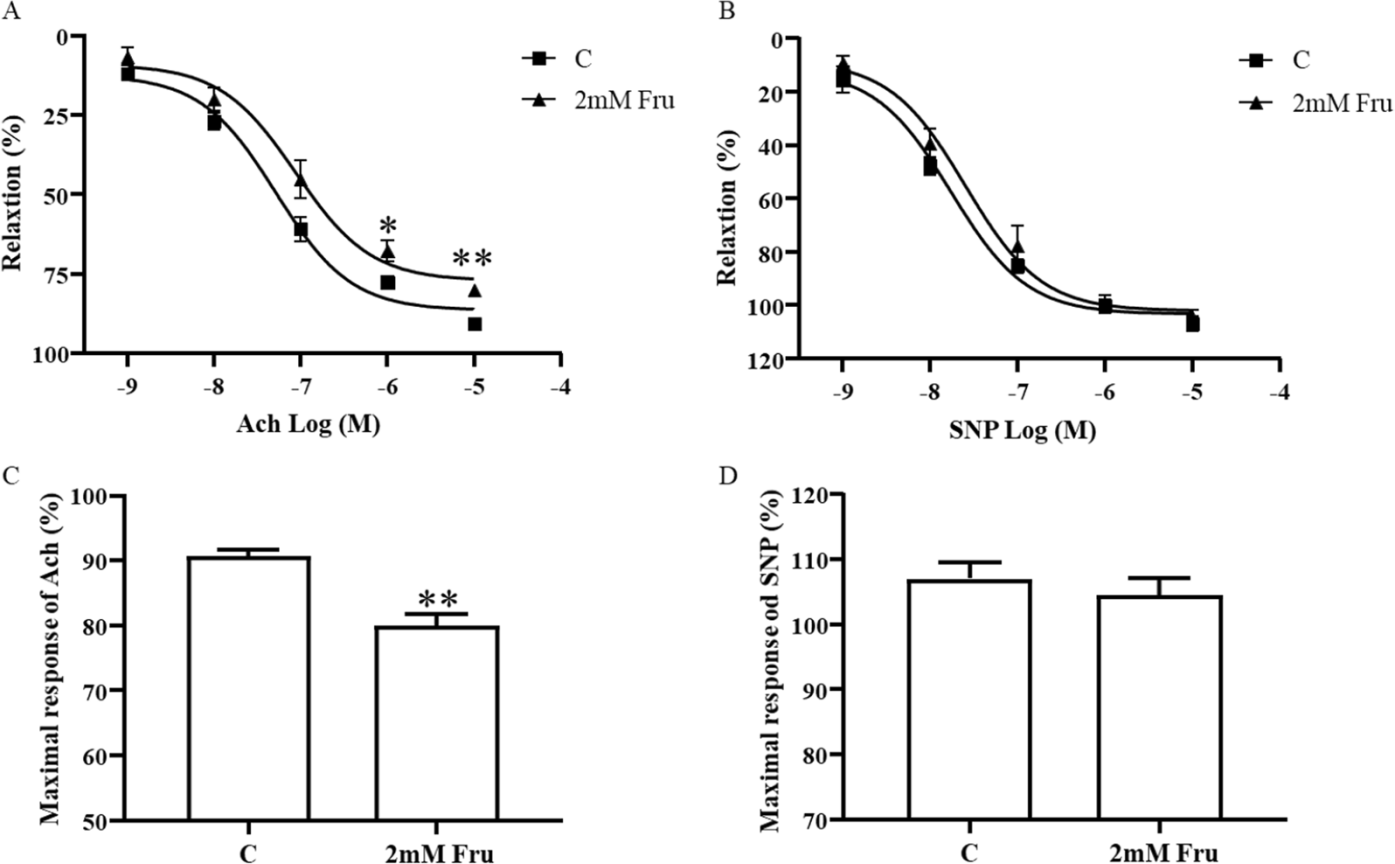
High fructose directly induces endothelium-dependent dysfunctional vasodilation. In each group of experiments, the aortic rings were stimulated by fructose (0.25 mM and 2 mM) for 0.5 h, precontracted by using 10^–6^ M PE, and relaxed by 10^–9^–10^–5^ M Ach or SNP. Wire myograph recording the effect of fructose on concentration–response curves to the endothelium-dependent dilator Ach (**A**) and the endothelium-independent dilator SNP (**B**) in aortic rings from C57BL/6 J mice (WT). **C, D** The maximal response of Ach and SPN. All data are presented as the mean ± SEM (n = 6 aortic rings). C, control; Fru, fructose; Ach, acetylcholine; SNP, sodium nitroprusside; PE, phenylephrine. *P < 0. 05, **P < 0. 01 versus C

Physiologically, although fructose is metabolized primarily in the liver, it also has a direct effect on nonliver tissues, especially blood vessels [32]. To determine the effect of a near-physiological dose of fructose on vasodilator function, we stimulated aortic rings from C57BL/6 J WT mice with 0.25 mM and 2 mM fructose for 0.5 h. Here, Ach could cause vasodilation by inducing endothelial cells to release NO, while SNP could directly release NO [33]. These two drugs can determine whether the blood vessel has endothelial-dependent dysfunction. Compared with the control (0.25 mM), high fructose (2 mM) significantly reduced the degree of aortic ring relaxation when the Ach concentration was 10^–6^ (*P* < 0.01) and 10^–5^ M (*P* < 0.01) (Fig. 1a). The relaxation responses to SNP were not different between the groups (Fig. 1b). The maximum response of these two diastolic drugs is visually displayed in Fig. 1c, d. These observations indicate that fructose could induce endothelium-dependent dysfunctional vasodilation.

### High fructose downregulates eNOS Ser177 phosphorylation resulting in a reduction in NO production

**Fig. 2:**
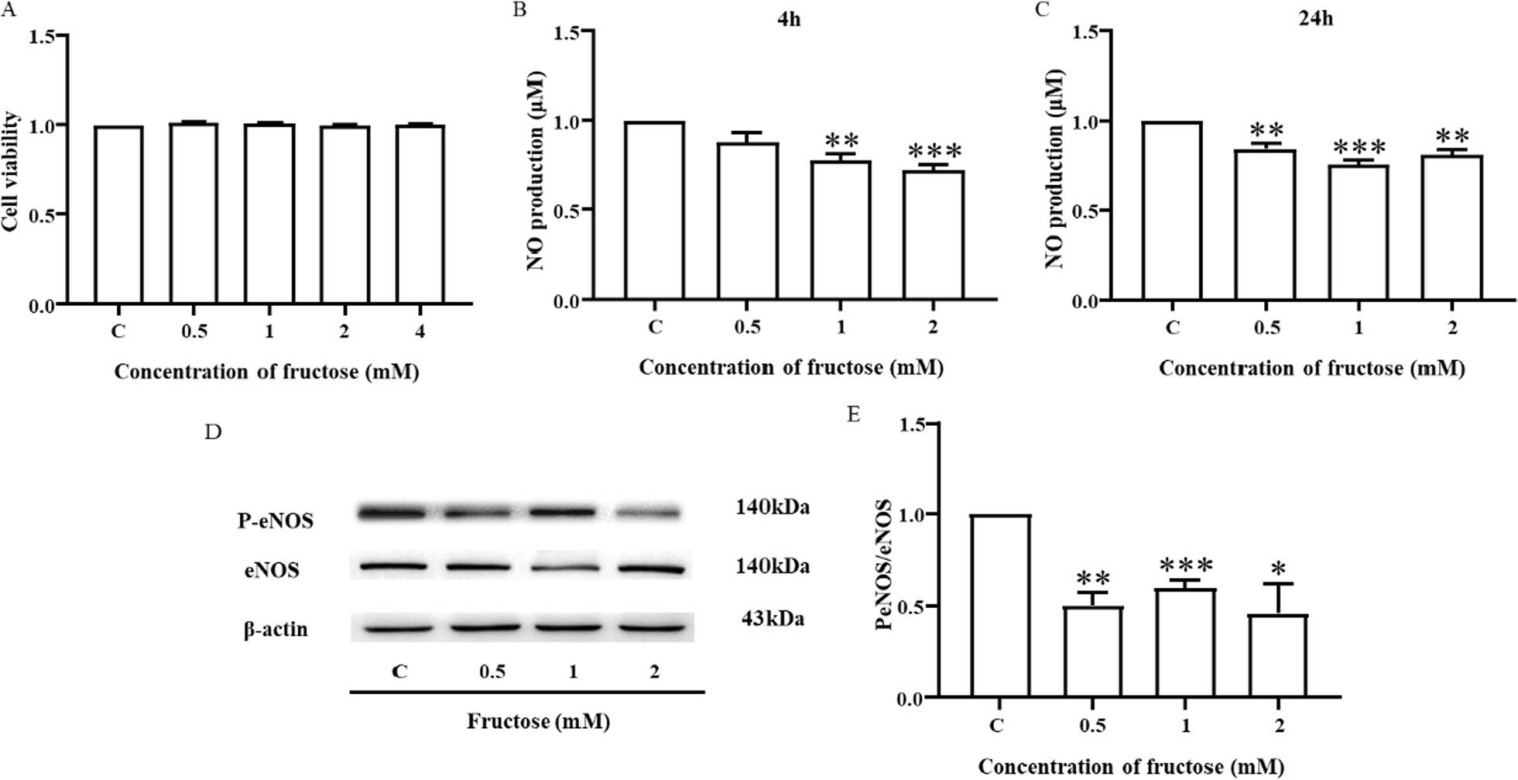
High fructose downregulates eNOS Ser177 phosphorylation resulting in a reduction in NO production. **A** Cell viability after stimulation with different concentrations of fructose for 24 h (n = 6). **B, C** NO production from MVECs after stimulation with fructose for 4 or 24 h (n = 4). **D, E** Western blot analysis showing the protein expression of eNOS and P-eNOS (Ser1177) in MVECs after stimulation with fructose for 8 h (n = 3). Relative protein levels of P-eNOS (Ser1177) were normalized to that of eNOS. All data are shown as the mean ± SEM. C, control. ^*^P < 0. 05, **P < 0. 01, ***P < 0.001 versus C

The ability of endothelial cells to produce NO is an indicator of vascular function [34]. The CCK-8 assay results showed that fructose in the range of 0.25–4 mM did not affect cell viability within 24 h (Fig. 2a), and follow-up experiments were performed with this concentration range. Different concentrations of fructose were used to stimulate MVECs for 4 or 24 h. NO production showed a decreasing trend with fructose concentration, which was significantly different compared with that of the control group (Fig. 2b, c). Next, MVECs were stimulated by fructose for 8 h. We noted that the phosphorylation level of eNOS at Ser1177 was markedly downregulated when fructose was administered at a dose higher than the control group (Fig. 2d, e).

### High fructose affects the eNOS pathway closely related to PP2A in endothelial cells

**Fig. 3:**
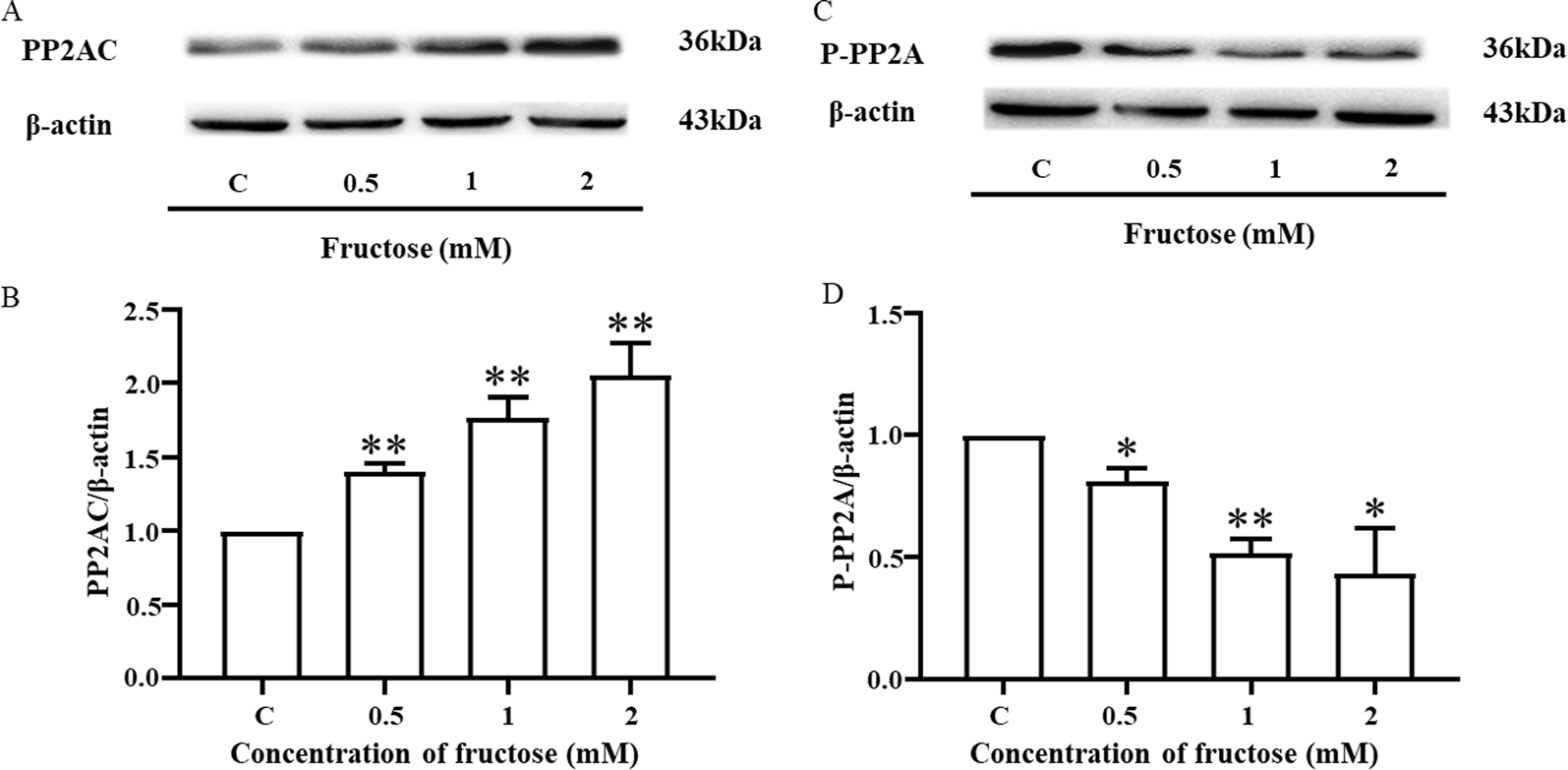
High fructose affects the eNOS pathway closely related to PP2A in endothelial cells. MVECs were stimulated by fructose for 8 h. **A, B** Western blot analysis showing the protein expression of PP2AC and **C, D** P-PP2A (Tyr307) in MVECs. Relative protein levels of PP2AC and P-PP2A (Tyr307) were normalized to that of β-actin. Data are presented as the mean ± SEM (n = 3). C, control. *P < 0. 05, **P < 0. 01 versus C

PP2A has been demonstrated to be an upstream factor regulating the phosphorylation level of eNOS [35]. With increasing fructose concentrations, the PP2AC protein expression increased significantly in MVECs (8 h) (Fig. 3a, b). Correspondingly, the phosphorylation level of PP2A at Tyr307 was decreased (Fig. 3c, d). The above results were statistically significant compared with those of the control group. Taken together, our results proved that fructose at or above twice the dose of the control could activate PP2A.

### PP2A inhibitor recovers high fructose-induced dysfunction mediated by NO reduction

**Fig. 4:**
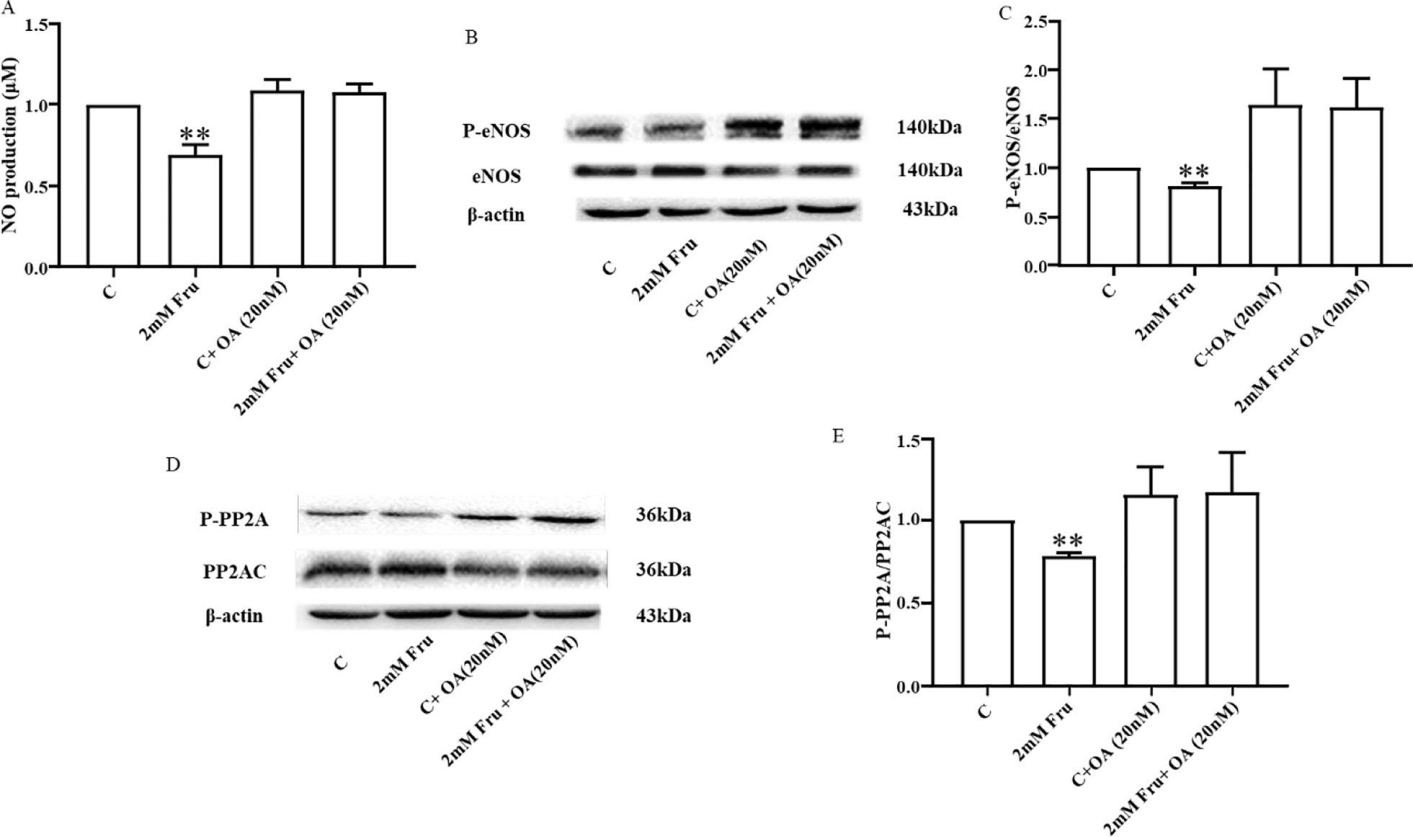
PP2A inhibitor recovers high fructose-induced dysfunction mediated by NO reduction. MVECs were pretreated with or without 20 nM OA for 1 h and then stimulated with fructose. **A** NO production from MVECs (after pretreatment with fructose for 24 h) (n = 4). Western blot analysis showing the protein expression of eNOS, P-eNOS (Ser1177) (**B, C**), PP2AC, and P-PP2A (Tyr307) (**D, E**) in MVECs (after pretreatment with fructose for 8 h) (n = 3). Relative protein levels of P-eNOS (Ser1177) were normalized to that of eNOS, and those of P-PP2A (Tyr307) were normalized to that of PP2AC. All data are presented as the mean ± SEM. C, control; Fru, fructose. *P < 0.05, **P < 0.01 when compared with the respective control groups

Subsequently, OA, a PP2A inhibitor was used to confirm the effect of fructose on PP2A/eNOS/NO for further investigation. The findings were consistent with the previous experimental results as 2 mM fructose significantly reduced the release of NO (Fig. 4a) and the levels of P-eNOS (Ser1177) (Fig. 4b, c) and P-PP2A (Tyr 307) compared with those of the control group (Fig. 4d, e). MVECs were pretreated with 20 nM OA for 1 h, and followed by administration of fructose for 8 h. The results showed that pretreatment with OA dramatically reversed the effect of high fructose on NO release and the expression levels of P-eNOS/eNOS and P-PP2A/PP2AC (Fig. 4a–e). These observations reveal the important role of fructose in regulating the PP2A/eNOS/NO pathway.

### Vasodilation function unchanged in the PP2A cKO mice under high fructose stimulation

**Fig. 5:**
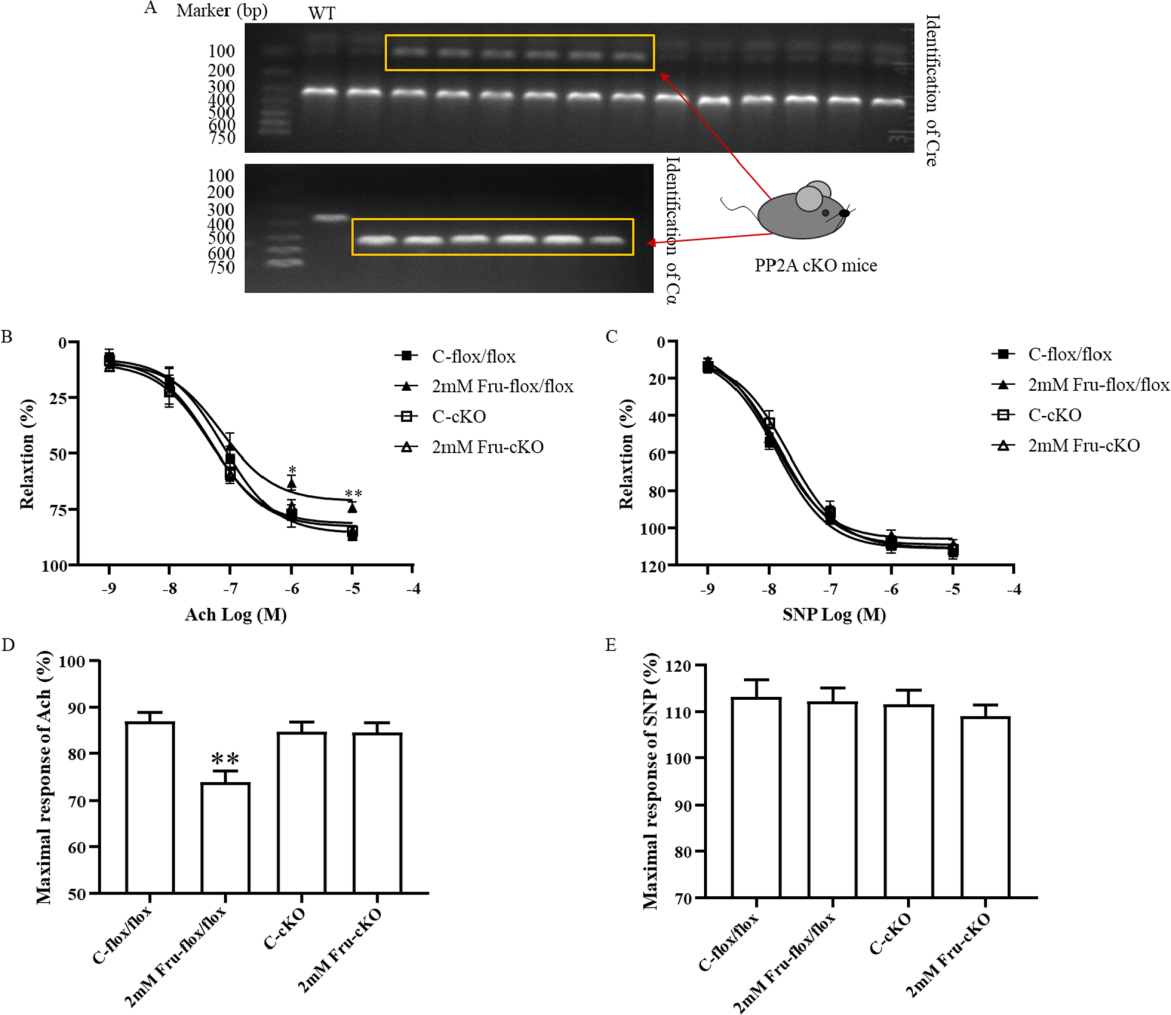
Vasodilation function unchanged in the PP2A cKO mice under high fructose stimulation. **A** Identification of the PP2ACα cKO mice by genotyping. In each group of experiments, the aortic rings were stimulated by fructose (0.25 mM and 2 mM) for 0.5 h, precontracted by using 10^–6^ M PE, and relaxed by 10^–9^–10^–5^ M Ach or SNP. Wire myograph recording the effect of fructose in concentration–response curves to the endothelium-dependent dilator Ach (**B**) and the endothelium-independent dilator SNP (**C**) in aortic rings from the PP2ACα^flox/flox^ and cKO mice. **D, E** The maximal response of Ach and SPN. All data are presented as the mean ± SEM (n = 5 aortic rings). C, control; Fru, fructose; Ach, acetylcholine; SNP, sodium nitroprusside; PE, phenylephrine. *P < 0. 05, **P < 0. 01 when compared with the respective control groups

To further elucidate the mechanisms controlling PP2A in fructose-induced endothelium-dependent dysfunctional vasodilation, we used conditional knockout mice in which the catalytic subunit Cα of PP2A synthase was specifically knocked out in the endothelium (PP2A cKO). We first identified the PP2ACα knockout gene. As shown in Fig. 5a, mice with bands at 100bq (identification of Cre) and 593bq (identification of Cα) were PP2A cKO mice. Consistent with the results of the WT mice, 2 mM fructose suppressed the Ach-induced vasodilation of the PP2ACα^flox/flox^ mice (Fig. 5b, d). Conditional knockout of the PP2ACα gene reduced dysfunctional vasodilation compared to that in the C-cKO group (Fig. 5b, d). Furthermore, the PP2A cKO mice showed a greater diastolic tendency at low concentrations of Ach than the PP2ACα^flox/flox^ mice, but there was no significant difference between them (Fig. 5b, d). The responses of thoracic aortic rings in each group to different concentrations of SNP were consistent (Fig. 5b, e), proving once again that the influence of fructose on this function was dependent on the endothelium rather than smooth muscle.

## Discussion

Fructose has been proven to induce endothelium-dependent dysfunctional vasodilation [30, 36]. However, the underlying mechanism remains largely unknown. Our present study demonstrated that fructose could downregulate the phosphorylation level of eNOS at Ser1177 by activating protein phosphatase, leading to a reduction in NO production, and ultimately resulting in dysfunctional vasodilation.

Some indicators of blood fructose concentration were reported in existing studies. According to reports, in humans, the serum fructose concentration after fasting could be as high as 0.1 mM, and the level increased to 0.5–2 mM after oral or intravenous administration [37, 38]. In experimental animals, the maximum concentration of fructose in the portal vein of rats could reach approximately 1 mM, and it drops to approximately 0.2 mM in the periphery [39]. After administration of a high fructose diet, the highest fructose concentration could also increase to 2 mM [40]. In mice, when no fructose is given, the serum fructose concentration in normal mice is approximately 0.25 to 0.3 mM, and it could increase to approximately 1.4 mM after being given a high fructose diet [31, 41]. Given the above, we used 0.25 mM fructose as the control and 2 mM fructose as the highest dose, so as to simulate the blood fructose concentration in vivo as much as possible in vitro experiments.

At present, the metabolic syndrome model induced by a high-fructose diet is widely used in research on related diseases [42], including vascular endothelial dysfunction [43]. We first observed the effect of fructose on vasodilation in isolated vessels from the WT mice. The blood vessel rings were less responsive to Ach and exhibited endothelium-dependent vasodilation dysfunction after preincubation with 2 mM fructose (Fig. 1). The results indicate that the effect of fructose on vasodilation is achieved by affecting endothelial cells and the NO pathway. Mahmoud and colleagues found that although the effect of fructose on Ach induced vasodilation was not statistically significant, the maximum diastolic rate in the fructose group was numerically approximately 15% lower than that in the control group, and the vasoconstrictive condition caused by increased KCl or PE also indicated the damaged caused by fructose to vascular reactivity (11 mM fructose vs. 11 mM glucose) [44]. Therefore, we focused on vascular endothelial cells in the following experiment.

NO is one of the most important endothelium-derived relaxing factors and is closely related to vasodilation [45]. Previous studies have shown that NO is released from vascular endothelial cells into smooth muscle cells, promoting the conversion of soluble guanosyl cyclase into cycloguanosyl monophosphate, and further realizing smooth muscle relaxation by reducing intracellular calcium concentration [27]. Our data show that the release of NO decreases with increasing fructose concentration without affecting cell activity (Fig. 2a–c). NO release from endothelial cells was correlated with eNOS phosphorylation. Phosphorylation sites that activate eNOS include Ser1177, Ser615 and Ser633 [29]. Ser1177 not only precisely regulates the production of NO but is also the first activated site of eNOS and thus can be regarded as the most important phosphorylation site [28, 29]. The degree of phosphorylation at Ser1177 was positively correlated with the flux of electrons through the reductase domain and the amount of NO released [46]. Phosphorylation of Ser615 alone is insufficient to stimulate the activity of eNOS, but this residue regulates eNOS activity by affecting phosphorylation of Ser1177 [47]. It has been reported that abnormal glucose metabolism in diabetes regulates eNOS function by inhibiting the phosphorylation of Ser1177, a process associated with Ser615 [48]. The phosphorylation of eNOS Ser633 started later than that of eNOS Ser1177 under vascular endothelial growth factor stimulation and shear stress [29]. Based on this background, we focused on the changes in Ser1177 in this study. In the present work, the phosphorylation level of eNOS at Ser1177 in the 0.5, 1, and 2 mM fructose groups was reduced and significantly different (Fig. 2d, e). Therefore, we further explored the effect of fructose based on the eNOS Ser 1177-NO axis.

The degree of eNOS phosphorylation is related to the phosphate dephosphorylation ability of phosphatase, in which PP2A plays an important role. PP2A is a whole enzyme complex family consisting of structural subunit A (PR65), regulatory subunit B, and catalytic subunit C (PP2AC) [49]. PP2AC has two isoforms: PP2ACα and PP2ACβ. The two isomers have very high homology in amino acid sequences, but PP2ACα transcripts levels are generally 10 times higher than PP2ACβ transcripts levels due to transcriptional regulation [50]. Studies have shown that the catalytic subunit PP2AC can undergo phosphorylation, methylation, acetylation, and other modifications after translation. The α isoform accounts for most intracellular serine/threonine phosphatase activity, and phosphorylation at its Tyr307 end could inactivate PP2A [51, 52], which further highlights the role of the α subtype. PP2A could separate the phosphate groups from the substrate by hydrolysing the phosphate bond, and dephosphorylating serine/threonine phosphate [53]. PP2A can affect the phosphorylation degree of many proteins in endothelial cells, such as occludin [54] and flotillin-1 (Ser315) [55]. eNOS is also one of the specific substrates for PP2A, and PP2A causes dephosphorylation of eNOS at Ser 1177 [56]. Our research found that this is indeed the case. The activation level of PP2A increased with increasing fructose concentration (Fig. 3). Moreover, we used 20 nM of the PP2A inhibitor OA to further determine the relationship between fructose and PP2A. OA, a compound originally extracted from sponges, specifically inhibits protein phosphatase 1 and PP2A, and it inhibits PP2A more efficiently than protein phosphatase 1 [57]. There was no significant difference in NO release or the expression levels of P-eNOS/eNOS and P-PP2A/PP2AC between the two groups after OA treatment (Fig. 4). Therefore, we speculate that fructose may activate PP2A to downregulate the phosphorylation level at Ser1177 of eNOS, resulting in a decrease in NO release. To further verify this hypothesis, we examined the effect of fructose on vasodilation in vitro in PP2ACα endothelial cKO mice. The results showed that fructose has no significant effect on vasodilatation function in the cKO mice (Fig. 5). We demonstrated that PP2A plays a key role in fructose-induced endothelium-dependent dysfunctional vasodilation. PP2A may be a potential target for the effective treatment of high fructose-induced endothelial dysfunction.

Our current data indicate that high fructose could induce endothelium-dependent dysfunctional vasodilation via PP2A-mediated eNOS Ser1177 dephosphorylation. However, this study has certain limitations. The effect of fructose on other phosphorylation sites that activate eNOS was not evaluated in this study. There are intramolecular interactions between Ser1177 and other major serine residues that may have profound implications for the overall accessibility of these residues [48]. The role of PP2A as a serine/threonine phosphatase in these residue interactions also requires further investigation. Therefore, the related mechanisms of fructose-induced vascular endothelial dysfunction in future studies could be based on this phenomenon.

## Conclusions

In conclusion, fructose could reduce the phosphorylation level of PP2ACα at Tyr307 to activate PP2A, and then downregulate the phosphorylation level of eNOS at Ser1177, resulting in a reduction in NO production, which leads to endothelium-dependent dysfunctional vasodilation. The results of this study may suggest novel targets to protect and improve endothelial function.

## Data Availability

The data used and/or analyzed during the current study are available from the corresponding author on reasonable request.

## Abbreviations

Ach: Acetylcholine
DMEM: Dulbecco's modified Eagle’s medium
DMSO: Dimethylsulfoxide
eNOS: Endothelial nitric oxide synthase
EDTA: Ethylene Diamine Tetraacetic Acid
NO: Nitric oxide
MVECs: Microvascular endothelial cells
OA: Okadaic acid
PP2A: Protein phosphatase 2A
PP2A cKO: PP2ACα endothelial-specific knockout
P-eNOS: Phosphorylated eNOS
PP2AC: Protein phosphatase 2A catalytic subunit
P-PP2A: Phosphorylated PP2AC
PE: Phenylephrine
PVDF: Polyvinylidene fluoride
SNP: Sodium nitroprusside
Ser: Serine
Tyr: Tyrosine
WT: Wild type
